# Oxidative Stress and Raloxifene

**DOI:** 10.5812/ijem.5341

**Published:** 2012-06-30

**Authors:** Gurkan Akgol, Arif Gulkesen, Salih Ozgocmen

**Affiliations:** 1 Bingol State Hospital, PMR Clinic, Bingol, Turkey; 2 Firat University, Faculty of Medicine, Deptartment PMR, Elazig, Turkey; 3 Erciyes University, Faculty of Medicine, Deptartment PMR, Division of Rheumatology, Kayseri, Turkey

**Keywords:** Raloxifene, Bone Metabolism

Dear Editor,

We read “Effects of raloxifene on bone metabolism in hemodialysis patients with type 2 diabetes” by Saito et al. (1) with a great interest. This paper shows that raloxifene works in diabetic or non-diabetic hemodialysis patients to reduce bone loss. This was shown by means of significant decrease in NTx and a significant increase in SOS measurements in both treatment groups compared to the un-treated control arms. We would like to draw attention to the possible anti-oxidant role of raloxifene regarding beneficial effects on the bone turnover markers as well as bone mass. The targeted population in this study consisted hemodialysis patients with type 2 diabetes who under a great oxidative stress related to both renal failure and diabetes (2, 3). We have previously demonstrated that women with post-menopausal osteoporosis had lower erythrocyte catalase (CAT) enzyme activity and higher erythrocyte malondialdehyde (MDA) levels (4). Interestingly, in another study we showed that raloxifene treatment for 3 months significantly enhanced CAT enzyme activity and reduced the MDA levels in women with PMO (5). Similar anti-oxidant effects of raloxifene were confirmed by others (6). Although neither discussed nor studied by means of enzymatic parameters, we would like to attract Authors’ attention to the potent anti-oxidant effect of raloxifene particularly in this special study population. Significant decrease in N-terminal cross-linking telopeptide of type I collagen (NTx) as well as oxidative stress parameters has been achieved with the use of potent anti-oxidants (lycopene) in patients with PMO (7). We think the results of this present study should also be admissible regarding the anti-oxidant effects of raloxifene particularly in hemodialysis patients with type 2 diabetes who are under great oxidative stress.
